# Concentrations of strontium, barium, cadmium, copper, zinc, manganese, chromium, antimony, selenium and lead in the equine liver and kidneys

**DOI:** 10.1186/2193-1801-3-343

**Published:** 2014-07-08

**Authors:** Nadine Paßlack, Barbara Mainzer, Monika Lahrssen-Wiederholt, Helmut Schafft, Richard Palavinskas, Angele Breithaupt, Konrad Neumann, Jürgen Zentek

**Affiliations:** Institute of Animal Nutrition, Department of Veterinary Medicine, Freie Universität Berlin, Königin-Luise-Str. 49, Berlin, 14195 Germany; Federal Institute for Risk Assessment, Max-Dohrn-Str. 8-10, Berlin, 10589 Germany; Institute of Veterinary Pathology, Department of Veterinary Medicine, Freie Universität Berlin, Robert-von-Ostertag-Str. 15, Berlin, 14163 Germany; Institute of Biometry and Clinical Epidemiology, Charité – Universitätsmedizin Berlin, Hindenburgdamm 30, Berlin, 12203 Germany

**Keywords:** Horses, Elements, Liver and kidneys, Feed, Environment

## Abstract

The concentrations of specific elements in the equine liver and kidneys are of practical relevance since horses are not only food-producing animals, but also partially serve as an indicator for the environmental pollution, as the basic feed includes plants like grass, grain and fruits. In this study, the concentrations of strontium (Sr), barium (Ba), cadmium (Cd), copper (Cu), zinc (Zn), manganese (Mn), chromium (Cr), antimony (Sb), selenium (Se) and lead (Pb) were measured in the liver, renal cortex and renal medulla of 21 horses (8 male; 13 female; aged between 5 months-28 years), using inductively coupled plasma mass spectrometry. Comparable Cu and Zn concentrations were detected in the liver and renal cortex, while approximately 50% lower concentrations were measured in the renal medulla. The lowest Sr, Cd and Se, but the highest Mn, Sb and Pb concentrations were measured in the liver. The Ba concentrations were comparable in the renal cortex and medulla, but lower in the liver of the horses. Gender-related differences were observed for Cd, Mn and Cr, with higher Cd concentrations in the liver, but lower Mn concentrations in the renal cortex and lower Cr concentrations in the renal medulla of female horses. Age-related differences were detected for most measured elements, however, the animal number per age-group was only low. In conclusion, the present study provides important reference data for the storage of Sr, Ba, Cd, Cu, Zn, Mn, Cr, Sb, Se and Pb in the liver and kidneys of horses, which are of practical relevance for an evaluation of the exposure of horses to these elements, either via feed or the environment.

**Electronic supplementary material:**

The online version of this article (doi:10.1186/2193-1801-3-343) contains supplementary material, which is available to authorized users.

## Introduction

In many countries, horses are food-producing animals and the storage of specific elements in the equine muscles and organs can become relevant for human and animal nutrition. The statistical office of the European Union (Eurostat) indicates that 214526 equids, including mainly horses, but also asses, mules and hinnies, were slaughtered in the European Union in the year 2007, with the highest rate in Italy (46.6%). In Germany, the per capita consumption of horse meat is low (0.04 kg), while other countries like Italy (1 kg), Luxembourg (0.68 kg) or France (0.35 kg) show a higher per capita consumption (Eurostat). Besides the relevance of horses for the food chain, these animals can partly be important as indicator for environmental pollution, as plants, the equine basic food resource, incorporate elements depending on the concentrations in the soil or air. For instance, horses are postulated to be an indicator for the cadmium (Cd) pollution of the environment (Elinder 1981). In this context, also other elements are of interest: the radioactive strontium (^90^Sr) is an important contaminant in soil, since it was released during overhead nuclear weapon tests in the 1950s and 1960s. The transition to plants and consecutively to food-producing animals and humans is intensively discussed since the 1960s. Beside Cd and Sr, antimony (Sb) is also interesting for an evaluation of the environmental exposition of horses. It has been reported (Daunderer 1998) that the air in Europe contains 8 ng Sb/m^3^ and the sea water 0.2 μg/l, while stream water contains 0.1-1 μg/l, and markedly higher concentrations (>1000 μg/kg dry matter) can be found in spruce needles, leaves of fruit-bearing trees and grass (Streit 1994). Moreover, the alkaline earth metal barium (Ba) is of interest. Ba shows a high reactivity and therefore exists in nature only in form of Ba compounds. Since only few information is available on the relevance of Ba for horses or other animals, the present study may help to generate new knowledge on its storage in the equine organs. Finally, lead (Pb) is an important environmental contaminant that can be ingested by water, soil and food (Brown and Margolis 2012). Since Pb accumulates in the organism, noxious effects can appear as a result of a high exposition (Ciobanu et al. 2012).

Beside the equine exposition to environmental contaminants, such as Cd, Sr, Sb, Ba and Pb, the intake of essential trace elements is of practical relevance. In particular, zinc (Zn), copper (Cu), chromium (Cr), manganese (Mn) and selenium (Se) should be considered. Since these elements can not only be provided by basic feed like hay or grain, but also by feed supplements, high individual variations in the element concentrations of equine organs can be assumed. However, considering that only few data on the Zn, Cu, Cr, Mn and Se concentrations in horses are available and that the corresponding studies were often performed several decades ago, further studies are needed to provide actual reference data. Moreover, since horses often reach a high age, a differentiation between several age-groups is reasonable, but also a differentiation between male and female horses, as a sexual dimorphism may occur for the accumulation of the elements in the equine organism.

The present study aimed at measuring the concentrations of Sr, Ba, Cd, Cu, Zn, Mn, Cr, Sb, Se and Pb in the liver, renal cortex and renal medulla of horses of different age and sex, providing actual data on the accumulation of these elements in typical storage organs. Therefore, inductively coupled plasma mass spectrometry (ICP-MS) was used. Since horses are also food-producing animals, the amounts of the elements in the liver and kidneys are of practical relevance, as these organs are edible animal by-products, used especially for animal nutrition, but also for humans.

## Material and methods

### Tissue samples and analysis

Liver and kidney samples of 10 euthanized horses were collected during routine post-mortem examinations in the Institute of Veterinary Pathology, Department of Veterinary Medicine, Freie Universität Berlin. Moreover, samples of 11 horses were collected in a local slaughterhouse (Roßschlächtermeister Frank Plaumann, Prenzlau, Germany). The euthanasia and the slaughtering were carried out according to the German Tierschutzgesetz (animal welfare act), while details on the drugs for the euthanasia are unspecified. The euthanasia and slaughtering were only based on medical purpose and were not performed to collect tissues for the present study or for the reason of other research. None of the authors participated in the euthanasia or slaughtering of the animals, but only obtained the samples in the Institute of Veterinary Pathology or directly in the slaughterhouse. The pathomorphological findings of the horses are summarized in Additional file 1: Table S1. The horses (8 male, 13 female) were aged between 5 months-28 years.

Before further analysis, all tissues were stored in polyethylene bags and frosted at -18°C. For the pathomorphological investigation, representative tissue samples were fixed in 4% neutral-buffered formaldehyde and embedded in paraffin. The samples were sectioned at 4 μm, mounted on glass slides and stained with haematoxylin and eosin (H&E).

### Preparation of the samples

The tissue samples were defrosted at room temperature, and 0.5 g of each sample was mixed with 5 ml concentrated nitric acid (65%) in a quartz vessel. This vessel was put into a Teflon® capsule, which was prepared with 5 ml bidistilled water and 2 ml hydrogen peroxide. Afterwards, a wet-chemical digestion was performed in a microwave (MLS GmbH, Leutkirch, Germany), where the Teflon® capsule was placed in a pressure vessel. The acid digestion lasted for 1 h, and maximum temperatures of 200°C were reached. After cooling, the samples were pipetted into graduated flasks that were provided with bidistilled water. The graduated flasks were filled up with bidistilled water to a total volume of 50 ml before the further measurement of the elements.

### Measurement of the elements

The concentrations of Sr, Ba, Cd, Cu, Zn, Mn, Cr, Sb, Se and Pb were measured using ICP-MS (model ELEMENT 1, Finnigan MAT, Bremen, Germany).

For quality assurance, the certified NBS SRM biological standard 1577 (bovine liver) (Spex Industries GmbH, Grasbrunn, Germany) was digested in the same way as all the samples and measured with the ICP-MS using the same method as for all samples. The results of the analyzed Certified Reference Material are presented in Table 1. For quality control (QC), the solution of a digested horse liver was used. The sample amount was 20 g digested in 20 parts and diluted in 1l water containing 6% nitric acid. The loop for the measurements was: blank, sample, sample, blank, QC-solution, sample, sample etc. During the entire series, the variation of the elements in the QC-solution was in between 10%. The detection limit (Table 2) was estimated from the mean of the blank, the standard deviation of the blank and some confidence factor.

**Table 1:** **Results (expressed in mg/kg) of the analyzed Certified Reference Material NBS SRM biological standard 1577 (bovine liver)**

	Certified value	Found
**Sr**	140 (not certified)	135 ± 9
**Ba**	0.94 (not certified)	0.89 ± 0.06
**Cd**	0.44 ± 0.06	0.48 ± 0.06
**Cu**	158 ± 7	156 ± 8
**Zn**	123 ± 8	120 ± 9
**Mn**	9.9 ± 0.8	9.5 ± 0.8
**Cr**	0.15 (not certified)	0.19 ± 0.06
**Sb**	0.0003 (not certified)	Not detected
**Se**	0.71 ± 0.07	0.69 ± 0.07
**Pb**	0.135 ± 0.015	0.132 ± 0.012

**Table 2:** **Limit of detection (**μ**g/kg) and limit of quantification (**μ**g/kg) in the present study**

	Limit of detection	Limit of quantification
**Sr**	0.007	0.021
**Ba**	0.007	0.021
**Cd**	0.056	0.168
**Cu**	0.053	0.159
**Zn**	0.041	0.123
**Mn**	0.077	0.231
**Cr**	0.056	0.168
**Sb**	0.006	0.018
**Se**	0.500	1.500
**Pb**	0.006	0.018

### Statistical analysis of the data

The data were analyzed using SPSS 15 (Chicago, USA). For the comparisons of the element concentrations in the liver, renal cortex and renal medulla of the horses, Friedman tests for dependent samples were performed. In case of a significant Friedman test, Wilcoxon signed rank tests were used to compare the element concentrations among the tissues (Table 3). For the evaluation of gender-related (Table 4) and age-related (Table 5) group differences (*P* < 0.05), the non-parametric Mann–Whitney-U-Test was performed. For the detection of the age-related differences, the horses were divided into different age groups. The dependence of the element concentrations in the liver, renal cortex and renal medulla on the sex and age (not classified) of the horses was examined (Table 6) using linear models with factor sex and covariate age (years). The level of significance was α = 0.05. Since the data were not normally distributed, the data are presented as median (minimum-maximum) in all tables.

**Table 3:** **Concentrations (**μ**g/kg) of strontium (Sr), barium (Ba), cadmium (Cd), copper (Cu), zinc (Zn), manganese (Mn), chromium (Cr), antimony (Sb), selenium (Se) and lead (Pb) in the liver and kidneys of horses (n = 21)**

	Liver		Cortex of the kidney		Renal medulla	
**Sr**	73.5	(23.5-283)^a^	170	(59.3-466)^b^	248	(40.7-1017)^c^
**Ba**	230	(107–427)^a^	340	(154–570)^b^	355	(179–982)^b^
**Cd**	798	(67.6-3700)^a^	8713	(315–42819)^b^	4647	(77.5-35091)^c^
**Cu**	4238	(2151–14609)^a^	4448	(2488–11393)^a^	1853	(1100–6511)^b^
**Zn**	21387	(8849–96681)^a^	21956	(12323–47110)^a^	12895	(8573–24653)^b^
**Mn**	1186	(515–3258)^a^	875	(344–1479)^b^	412	(242–1292)^c^
**Cr**	116	(22.0-427)	84.7	(16.3-514)	91.5	(27.6-348)
**Sb**	14.8	(0.00-23.8)^a^	10.8	(0.00-13.5)^b^	10.8	(0.00-12.0)^c^
**Se**	131	(69.6-336)^a^	739	(356–1599)^b^	255	(87.5-647)^c^
**Pb**	82.3	(0.00-301)^a^	34.0	(0.00-118)^b^	18.2	(0.00-103)^c^

Differences (*P* < 0.05) within a row are marked with different superscript letters.

**Table 4:** **Concentrations (**μ**g/kg) of strontium (Sr), barium (Ba), cadmium (Cd), copper (Cu), zinc (Zn), manganese (Mn), chromium (Cr), antimony (Sb), selenium (Se) and lead (Pb) in the liver and kidneys of horses, depending on the sex of the animals (♂: n = 8; ♀: n = 13)**

	Liver				Cortex of the kidney				Renal medulla			
	♂		♀		♂		♀		♂		♀	
**Sr**	80.3	(26.3-135)	67.7	(23.5-283)	185	(59.3-251)	168	(62.8-466)	228	(87.6-360)	295	(40.7-1017)
**Ba**	233	(214–271)	230	(107–427)	397	(240–570)	323	(154–508)	366	(221–564)	355	(179–982)
**Cd**	475	(67.6-3669)^a^	1074	(551–3700)^b^	7522	(315–42819)	13967	(3048–36562)	1776	(77.5-17500)	5099	(489–35091)
**Cu**	5181	(2871–14609)	4236	(2151–7966)	4806	(3009–10732)	4448	(2488–11393)	1960	(1131–5331)	1842	(1100–6511)
**Zn**	22063	(8849–96681)	21387	(12579–45690)	22779	(13645–47110)	21956	(12323–44963)	11624	(9054–24653)	12990	(8573–21854)
**Mn**	2345	(515–3258)	992	(544–2588)	1261	(564–1479)^a^	805	(344–1268)^b^	540	(248–1292)	372	(242–744)
**Cr**	119	(52.3-427)	76.6	(22.0-178)	100	(16.3-188)	44.8	(17.0-514)	106	(84.4-348)	82.7	(27.6-175)
**Sb**	15.3	(0.00-21.6)	0.35	(0.00-23.8)	11.7	(0.00-12.5)	0.00	(0.00-13.5)	11.0	(0.00-11.6)	0.00	(0.00-12.0)
**Se**	130	(69.6-319)	151	(71.0-336)	771	(462–1197)	683	(356–1599)	264	(150–647)	185	(87.5-522)
**Pb**	70.6	(25.9-158)	90.7	(0.00-301)	37.2	(0.00-114)	32.6	(0.00-118)	23.2	(0.00-40.5)	16.1	(0.00-103)

Group comparisons were separately calculated for each element and each tissue. Differences (*P* < 0.05) within a row are marked with different superscript letters.

**Table 5:** **Concentrations (**μ**g/kg) of strontium (Sr), barium (Ba), cadmium (Cd), copper (Cu), zinc (Zn), manganese (Mn), chromium (Cr), antimony (Sb), selenium (Se) and lead (Pb) in the liver and kidneys of horses, depending on the age of the animals** ^**1**^

	Age	Liver		Cortex of the kidney		Renal medulla	
**Sr**	**0-1**	77.2	(26.3-121)	179	(59.3-251)	228	(87.6-328)
	**2-4**	66.4	(40.1-92.8)	153	(84.3-222)	164	(110–218)
	**5-8**	73.5	(39.3-80.7)	168	(114–318)	409	(303–1017)
	**9-13**	45.5	(23.5-135)	136	(62.8-326)	257	(40.7-609)
	**16-28**	100	(48.0-283)	199	(167–466)	257	(168–439)
**Ba**	**0-1**	229	(214–256)	388	(240–570)	366	(221–485)
	**2-4**	243	(214–271)	375	(261–489)	374	(287–460)
	**5-8**	230	(107–236)	297	(265–367)	355	(248–982)
	**9-13**	242	(117–280)	331	(154–461)	328	(179–564)
	**16-28**	228	(154–427)	380	(252–508)	346	(302–443)
**Cd**	**0-1**	263	(67.6-611)^a^	3065	(315–8713)^a^	294	(77.5-6800)^ac^
	**2-4**	475	(460–490)^a^	8167	(7412–8922)^ab^	1776	(1429–2123)^ad^
	**5-8**	743	(551–1577)^ab^	6606	(5729–8781)^ab^	1141	(738–2646)^ad^
	**9-13**	976	(699–3669)^b^	14312	(7633–42819)^ab^	6031	(3422–17500)^bc^
	**16-28**	1536	(798–3700)^b^	24027	(3048–36562)^b^	9273	(489–35091)^bd^
**Cu**	**0-1**	6057	(5609–14609)^a^	5995	(4347–10732)	1960	(1821–5331)^ab^
	**2-4**	3473	(2871–4074)^ab^	3947	(3709–4185)	1469	(1131–1807)^ab^
	**5-8**	3172	(2151–4613)^b^	3689	(2488–6250)	1295	(1100–1842)^a^
	**9-13**	3872	(2926–7966)^ab^	5960	(3009–11393)	2182	(1542–6511)^b^
	**16-28**	4281	(2403–5445)^b^	3996	(3194–5311)	2108	(1360–3421)^ab^
**Zn**	**0-1**	30609	(16724–96681)	33881	(18866–47110)	10902	(9230–17235)^ab^
	**2-4**	18218	(17781–18654)	18020	(16408–19633)	10304	(9054–11554)^ab^
	**5-8**	14857	(14790–22329)	20553	(15948–22537)	10406	(8573–12449)^a^
	**9-13**	26164	(8849–45690)	25820	(13645–44963)	16808	(11056–24653)^b^
	**16-28**	21675	(12579–38140)	21571	(12323–29907)	14390	(8935–21854)^ab^
**Mn**	**0-1**	2727	(2252–3258)^a^	1281	(609–1479)	501	(288–1292)
	**2-4**	1780	(1113–2447)^ab^	926	(564–1289)	433	(248–618)
	**5-8**	1186	(976–1877)^b^	711	(542–1035)	350	(290–363)
	**9-13**	824	(515–1560)^b^	889	(522–1246)	521	(318–1108)
	**16-28**	1580	(544–2588)^ab^	847	(344–1268)	403	(242–744)
**Cr**	**0-1**	137	(116–427)^a^	127	(16.3-188)	97.5	(91.2-188)
	**2-4**	87.3	(52.3-122)^ab^	61.1	(40.7-81.5)	100	(84.4-116)
	**5-8**	42.3	(40.5-145)^ab^	36.5	(28.1-107)	40.1	(27.6-103)
	**9-13**	67.1	(22.0-118)^b^	64.7	(17.0-122)	121	(38.7-348)
	**16-28**	140	(34.2-178)^ab^	96.9	(19.4-514)	86.3	(27.9-119)
**Sb**	**0-1**	15.3	(0.00-21.6)	11.7	(0.00-12.4)	11.0	(0.00-11.5)
	**2-4**	10.5	(0.00-20.9)	6.05	(0.00-12.1)	5.67	(0.00-11.3)
	**5-8**	0.00	(0.00-14.8)	0.00	(0.00-12.0)	0.00	(0.00-11.6)
	**9-13**	0.00	(0.00-18.5)	0.00	(0.00-12.5)	0.00	(0.00-11.6)
	**16-28**	17.1	(0.00-23.8)	11.1	(0.00-13.5)	10.9	(0.00-12.0)
**Se**	**0-1**	130	(109–201)^ab^	771	(539–1088)	213	(150–647)^a^
	**2-4**	180	(120–240)^ab^	896	(721–1070)	253	(234–273)^ab^
	**5-8**	72.6	(71.0-180)^a^	544	(468–1599)	95.2	(87.5-124)^b^
	**9-13**	130	(69.6-319)^ab^	516	(356–1197)	246	(147–542)^a^
	**16-28**	271	(99.3-336)^b^	834	(400–981)	325	(171–522)^a^
**Pb**	**0-1**	70.6	(62.5-136)	37.2	(0.00-114)	23.9	(0.00-33.5)
	**2-4**	91.8	(25.9-158)	32.3	(8.17-56.5)	17.4	(6.51-28.2)
	**5-8**	82.3	(0.00-90.7)	25.9	(0.00-84.7)	16.1	(0.00-21.0)
	**9-13**	110	(45.1-170)	21.1	(6.76-77.2)	10.0	(2.48-40.5)
	**16-28**	106	(0.00-301)	35.2	(0.00-118)	22.1	(0.00-103)

^1^n = 4 (0–1 years), n = 2 (2–4 years), n = 3 (5–8 years), n = 6 (9–13 years), n = 6 (16–28 years).

Group comparisons were separately calculated for each element and each tissue. Differences (*P* < 0.05) within a column are marked with different superscript letters.

**Table 6:** **Dependence of the storage of strontium (Sr), barium (Ba), cadmium (Cd), copper (Cu), zinc (Zn), manganese (Mn), chromium (Cr), antimony (Sb), selenium (Se) and lead (Pb) in the liver and kidneys of horses (n = 21) on the sex and age of the animals (** ***P*** **-values)**

	Liver		Cortex of the kidney		Renal medulla	
	**Sex**	**Age**	**Sex**	**Age**	**Sex**	**Age**
**Sr**	0.350	0.128	0.890	0.289	0.211	0.701
**Ba**	0.388	0.331	0.129	0.420	0.809	0.721
**Cd**	0.997	0.158	0.652	**0.039**	0.982	0.214
**Cu**	0.569	0.413	0.575	0.156	0.786	0.593
**Zn**	0.908	0.259	0.940	0.276	0.318	0.138
**Mn**	0.151	0.843	**0.026**	0.465	0.135	0.692
**Cr**	0.363	0.997	0.567	0.395	**0.015**	0.121
**Sb**	0.379	0.550	0.356	0.562	0.393	0.596
**Se**	0.145	**0.012**	0.345	0.557	0.077	0.128
**Pb**	0.745	0.961	0.704	0.996	0.688	0.438

## Results

### Total concentrations of Sr, Ba, Cd, Cu, Zn, Mn, Cr, Sb, Se and Pb in the liver, cortex of the kidney and renal medulla

The detection of the elements in the liver and kidney of the horses revealed large individual differences (Table 3). The Sr concentrations were the lowest in the liver (73.5 (23.5-283) μg/kg), followed by the renal cortex (170 (59.3-466) μg/kg) and the renal medulla (248 (40.7-1017) μg/kg) (*P* < 0.05). In contrast, the highest concentrations of Mn and Sb were detected in the liver compared to the cortex or medulla of the kidneys (*P* < 0.05). Cu and Zn were the lowest in the renal medulla when compared to the liver and the renal cortex (*P* < 0.05). The measured hepatic Ba concentrations amounted to 230 (107–427) μg/kg, while medians between 340–355 μg/kg were measured in the kidney (*P* < 0.05). The highest Cd and Se concentrations were detected in the renal cortex of the horses, followed by the renal medulla and the liver (*P* < 0.05). Markedly higher Pb concentrations were detected in the liver (82.3 (0.00-301) μg/kg) of the horses when compared to the renal cortex (34.0 (0.00-118) μg/kg) and the renal medulla (18.2 (0.00-103) μg/kg) (*P* < 0.05).

### Gender-specific differences

The sex of the horses had only minor impact on the concentrations of the measured elements in the liver and kidney of the horses (Table 4). Higher concentrations of Cd were found in the liver of female horses (1074 (551–3700) μg/kg) compared to male horses (475 (67.6-3669) μg/kg; *P* < 0.05). Moreover, male horses showed higher concentrations of Mn in the renal cortex (1261 (564–1479) μg/kg) compared to female horses (805 (344–1268) μg/kg; *P* < 0.05). The linear model also detected a gender-dependent difference (*P* = 0.015) for the storage of Cr in the renal medulla, where lower concentrations were found in female horses (82.7 (27.6-175) μg/kg) compared to male horses (106 (84.4-348) μg/kg).

### Age-related differences

The age-related differences in the element concentrations in the liver and kidney of the horses are presented in Table 5. In particular, the Cd concentrations varied depending on the age of the animals, with lower concentrations in the liver of young horses (0–4 years) compared to older horses (9–28 years). Similar results were observed in the renal cortex, where markedly lower Cd concentrations (3065 (315–8713) μg/kg) were measured in horses up to 1 year compared to horses between 16–28 years (24027 (3048–36562) μg/kg) (*P* < 0.05). The Cd concentrations were also lower in the renal medulla of younger horses than in older horses, demonstrating differences (*P* < 0.05) when comparing the age group 0–1 year with the group 16–28 years and also horses between 2–8 years with 9–13 years old horses.

The Cu concentrations in the liver were the highest in horses up to 1 year (6057 (5609–14609) μg/kg), but lower (*P* < 0.05) in horses between 5–8 (3172 (2151–4613) μg/kg) and 16–28 years (4281 (2403–5445) μg/kg). In the renal medulla, the Cu concentrations were higher in horses between 9–13 years (2182 (1542–6511) μg/kg) compared to horses between 5–8 years (1295 (1100–1842) μg/kg) (*P* < 0.05). Similar results were observed for Zn, where the concentrations in the renal medulla were 60% higher in horses between 9–13 years compared to horses between 5–8 years (*P* < 0.05).

The hepatic Mn and Cr concentrations were higher (*P* < 0.05) in very young horses (0–1 year) when compared to older horses (9–13 years), and for Mn additionally when compared to middle-aged horses (5–8 years).

Horses between 5–8 years showed lower Se concentrations compared to horses between 16–28 years (liver) or compared to horses between 0–1 years and 9–28 years (renal medulla) (*P* < 0.05).

When the age of the horses was not classified (Table 6), a dependence for the storage of Se (liver) and Cd (renal cortex) on the age of the horses was detected (*P* < 0.05).

## Discussion

This study provides new data on the storage of Sr, Ba, Cd, Cu, Zn, Mn, Cr, Sb, Se and Pb in the liver and kidneys of horses, considering the different age and the sex of the animals. As only few literature is available, the present data can be considered as important reference data, but also give some indication of the exposure of horses to specific elements in the environment or feed.

Although the background of the horses is not completely known, it can be assumed that the animals came from different areas of Germany. Since no fattening farms exist in Germany (Gudehus 2006), the origin of slaughtered horses largely differ. In particular, age and field of use (e.g. sports or leisure riding, reproduction) of the horses vary (Gudehus 2006). The horses chosen for the present investigation might therefore represent the situation regarding the origin of meat producing horses in Germany. However, the observed individual variations in the element concentrations in the equine liver and kidneys indicate differences in the exposure of the horses to environmental contaminants and dietary mineral supply. With regard to the use of equine by-products in the food chain, variations in the uptake of specific environmental contaminants and trace elements by the ingestion of equine organs can be assumed and should be considered for risk assessment in human and animal nutrition.

As a first interesting result, Cu was stored in approximately identical amounts in the liver (4238 (2151–14609) μg/kg) and renal cortex (4448 (2488–11393) μg/kg) of the horses. The liver is considered to be a storage organ for Cu (Plumlee and Johnson 1996), and comparable amounts of Cu were also measured in the equine liver in a previous study (Hecht 1984). However, the Cu concentrations in the renal cortex and renal medulla (1853 (1100–6511) μg/kg) observed in the present study were lower than previously measured in the renal cortex of female (7300 μg/kg) and male (7500 μg/kg) horses (Koizumi et al. 1989). No gender-related differences were observed for the storage of Cu in the present study, and only small age-dependent variations were detected, with the highest hepatic Cu concentrations in foals up to 1 year and lower (*P* < 0.05) concentrations in middle-aged (5–8 years) and old (16–28 years) horses. In the renal medulla, lowest Cu concentrations were measured in horses between 5–8 years and highest in horses between 9–13 years. These findings are only partly in accordance with further data from horses (Plumlee and Johnson 1996), where no gender- or age-related differences were detected for the Cu concentrations in the equine liver and kidneys. In general, the storage of Cu in the organism depends on the amount, kind and duration of Cu supplementation, and the concentration of protein, Zn and iron in the diet. Moreover, the status of the animal and the used feed additives are important factors (Prothmann 1975). In this context, recent data from cattle (Miranda et al. 2006a; Waegeneers et al. 2009) indicate dietary differences between horses and cattle, but also differences in the storage of Cu in the organism. In these studies (Miranda et al. 2006a; Waegeneers et al. 2009), marked higher hepatic Cu concentrations were found in cattle (34.3 (1.86-181) mg/kg and 80.1 ± 66.7 mg/kg, respectively), while the renal Cu concentrations were comparable to the presently measured Cu concentrations in the renal cortex of the horses. Unfortunately, the present study design did not allow an estimation of the feeding regime of the horses. Further studies are required to explain the detected inter- and intra-species related differences.

Similar to the results on the Cu concentrations, Zn was also equally stored in the liver and the renal cortex of the horses. No differences were observed depending on the sex of the animals, which is in accordance with further studies (Hecht 1986; Koizumi et al. 1989). While Hecht (1986) could not find an age-dependency for the storage of Zn in the equine liver and kidney, the present study could also demonstrate only one group difference for the Zn concentrations in the renal medulla of the horses, with lower amounts in horses between 5–8 years compared to horses between 9–13 years. In general, the measured hepatic Zn concentrations (21387 (8849–96681) μg/kg) were approximately 50% lower compared to data from previous studies in horses (Kowalczyk et al. 1986; Koizumi et al. 1989) and cattle (Miranda et al. 2006a; Waegeneers et al. 2009). The detected Zn concentrations in the cortex (21956 (12323–47110) μg/kg) and medulla (12895 (8573–24653) μg/kg) of the kidneys were in accord with previous results in horses (Koizumi et al. 1989) and cattle (Miranda et al. 2006a; Waegeneers et al. 2009), while another study found markedly higher Zn concentrations in the equine liver (88100–109000 μg/kg) and kidneys (37700–52900 μg/kg) (Hecht 1984). For the interpretation of the present results, it should be taken into consideration that the Zn accumulation in the equine organism might partly depend on the environmental pollution. Farmer and Farmer (2000) evaluated the Zn concentrations in the liver and kidneys of horses in a metal processing region in eastern Kazakhstan and found markedly higher Zn values in the equine liver (37.73 ± 3.95 mg/kg in the district Tavricheskiy and 63.28 ± 4.33 mg/kg in the district Samarskiy) and in the equine kidneys (36.05 ± 4.43 mg/kg in the district Tavricheskiy and 49.39 ± 4.03 mg/kg in the district Samarskiy) than in the present study or in the previous studies of Kowalczyk et al. (1986) and Koizumi et al. (1989).

The Mn concentrations were the highest in the liver of the horses, followed by the renal cortex and the renal medulla. In general, female horses stored less Mn in the organs compared to male horses, however, this difference was only statistically significant in the renal cortex. Considering the age of the animals, the hepatic Mn concentrations were higher in foals (0–1 year) compared to middle-aged horses (5–13 years). No age-effect was observed for the Mn concentrations in the cortex and medulla of the kidneys. Partially, these findings contrast with observations in humans, where a gender- and age-dependency was found for the storage of Mn in the organism (Anke et al. 1978). The authors reported that women showed higher Mn concentrations in the rips and kidneys compared to men and that the Mn concentrations decreased with age. However, another study could not demonstrate an age-dependency for the Mn accumulation in humans (Schroeder et al. 1966). The higher Mn concentrations in foals compared to older horses, as observed in the present study, are in accordance with findings in cats, where the highest Mn concentrations in the liver and bones were found in kittens at an age of 4 weeks resp. 8–12 weeks compared to adult cats (Stratmann 1988).

Highest Se concentrations were found in the renal cortex (739 (356–1599) μg/kg), while markedly lower concentrations were detected in the renal medulla (255 (87.5-647) μg/kg) and the liver (131 (69.6-336) μg/kg) of the horses. The measured Se concentrations are comparable to data reported in cattle (Salisbury et al. 1991). The Se concentrations were lower in horses between 5–8 years when compared to horses between 16–28 years (liver) or when compared to horses between 0–1 and 9–28 years (renal medulla). These findings are in contrast to data of Teilmann and Hansen (1984), where no age-dependency was observed for the Se concentrations in the kidneys of horses. In general, the main natural source of Se for horses are plants, while the amounts of Se differ depending on the Se concentrations in the soil (Zhu et al. 2009). An excess of Se, either in seleniferous regions or by an oversupplementation, can result in toxicosis (Coenen 2013). Desta et al. (2011) reported that horses showed toxic Se concentrations in the liver (6.13 ± 0.31 mg/kg) and in the kidneys (6.25 ± 0.3 mg/kg), probably resulting from an injection of a toxic Se preparation.

Cd was mainly stored in the kidneys of the horses, with the highest concentrations in the renal cortex (8713 (315–42819) μg/kg), followed by the renal medulla (4647 (77.5-35091) μg/kg) and the liver (798 (67.6-3700) μg/kg). The amounts were always higher in female horses compared to male horses, however, this difference was only statistically significant for the hepatic Cd concentrations. The higher Cd accumulation in female horses is in accord with previous studies in horses (Baldini et al. 2000) and humans (Kjellström 1979). The analysis of age-related differences demonstrated that young horses showed lower amounts of Cd in the liver and kidneys compared to older horses. This finding is in accordance with further data from horses (Baldini et al. 2000) and cattle (Rudy 2009), however, other authors found an increase of Cd with increasing age of horses (Salmi and Hirn 1981) and humans (Anke et al. 1978), but a subsequent decrease at an older age. In general, the presently measured hepatic Cd concentrations were lower than further results in horses (Salmi and Hirn 1981; Baldini et al. 2000; Farmer and Farmer 2000), and also lower Cd concentrations were found in the kidneys of the horses when compared to previous data (Elinder et al. 1981; Salmi and Hirn 1981; Junnila et al. 1987; Baldini et al. 2000; Farmer and Farmer 2000; Beldomenico et al. 2001). Interestingly, the measured Cd concentrations in the livers and kidneys of the horses were markedly higher compared to cattle (Rudy 2009; Waegeneers et al. 2009).

An explanation for the lower Cd concentrations in the equine liver and kidneys demonstrated in the present study compared to the results of previous studies in horses could possibly be found in the environmental load, since it was assumed that horses can be considered as an indicator for the Cd pollution (Elinder 1981; Farmer and Farmer 2000). The higher Cd concentrations in the organs of horses compared to cattle might be explained by the higher age at the time of slaughtering.

Cd concentrations in the food chain are of current interest for risk assessment. The EFSA Scientific Panel on Contaminants in the Food Chain (2004a) has indicated that the ingestion of liver and kidneys of horses, ruminants and wildlife can considerably contribute to human overall Cd exposure. According to Regulation (EC) No 1881/2006 latest amended by Regulation (EC) No 629/2008, the maximum level for Cd in liver of horses is 0.50 mg/kg fresh weight, and the maximum level for Cd in kidneys of horses 1.0 mg/kg fresh weight. Both maximum levels have been exceeded in the present study when the median values were considered. The EFSA Panel on Contaminants in the Food Chain (2009) indicates that 3.7% of liver samples (bovine, sheep, pig, poultry, horse) and 0.96% of kidney samples (bovine, sheep, pig, poultry, horse) have exceeded the maximum levels for Cd. The present data underline the relevance of equine organs in the food chain for human Cd exposure and confirm the opinion of the EFSA Panel on Contaminants in the Food Chain (2009; 2011) that the Cd exposure of humans should be reduced.

The Sr concentrations were the highest in the renal medulla, followed by the renal cortex and the liver. No gender- or age-dependent differences were detected. This is in contrast to findings in humans, where newborns and children up to 9 years showed higher Sr concentrations in the kidneys and liver compared to adults, and persons older than 50 years accumulated increased amounts of Sr in the kidneys (Schroeder et al. 1972). Possible explanations for these variations may include differences in the living conditions or basic food resource, which needs further evaluation in future studies.

The Ba concentrations in the liver of the horses (230 (107–427) μg/kg) were lower when compared to the renal cortex (340 (154–570) μg/kg) and the renal medulla (355 (179–982) μg/kg). No age- or gender-related differences could be detected for the hepatic and renal Ba concentrations. This is in contrast to findings in humans, where the Ba concentrations in the liver and kidneys of children were higher compared to the amounts in the organs of older persons (Schroeder et al. 1972). The authors also indicated that the geographic background of humans is important for the storage of Ba in the organism.

Pb was mainly accumulated in the liver (82.3 (0.00-301) μg/kg) of the horses when compared to the cortex (34.0 (0.00-118) μg/kg) and medulla (18.2 (0.00-103) μg/kg) of the kidneys, while no age- or gender-related differences were observed. This observation is in contrast to data of cattle, where increased hepatic Pb concentrations were detected with age (Rudy 2009). In this study (Rudy 2009), the Pb concentrations in the liver of cattle (0.125-0.179 mg/kg) were higher than presently measured in the equine organs, while other authors (Waegeneers et al. 2009) found comparable amounts in the bovine liver (0.082 ± 0.126 mg/kg), but higher Pb concentrations in the bovine kidneys (0.212 ± 0.209 mg/kg). In this context, it should not go unmentioned that Blanco-Penedo et al. (2006) found a positive correlation between the hepatic Cu and Pb accumulation in cattle, why the authors suggest an interaction of these elements after ingestion. Further studies should evaluate the metabolism of these two elements and the potential impact on hepatic storage, not only in the bovine, but also in the equine organism.

As Pb is an important environmental contaminant, its concentrations in the equine organs may depend on the exposure of the animals. Horses in a metal processing region in eastern Kazakhstan (Farmer and Farmer 2000) showed markedly higher Pb concentrations in the liver (1.29-2.22 mg/kg) and kidneys (0.84-1.27mg/kg) compared to the horses of the present study. The hepatic (0.75-1.02 mg/kg) and renal (0.72-1.03 mg/kg) Pb concentrations were also higher in cattle of this metal processing region. When compared to previous data from horses in Central Europe (Kośla et al. 1989), the presently measured Pb concentrations in the equine organs were lower than 25 years ago (1.8 ± 1.7 mg/kg dry matter of the liver and 1.2 ± 0.72 mg/kg dry matter of the kidney). This observation may indicate a reduced environmental Pb pollution and therefore a reduced health risk for horses, but also for humans in Central Europe. The EFSA Scientific Panel on Contaminants in the Food Chain (2004b) concluded that food of animal origin is not the main source for human Pb exposure. However, variations may depend on environmental pollution. In this context, reports on lead poisoning exist for horses and other livestock in contaminated areas (e.g. Levine et al. 1976; Palacios et al. 2002; Lemos et al. 2004; Miranda et al. 2006b; Rodríguez-Estival et al. 2012). In 2010, the EFSA Panel on Contaminants in the Food Chain (CONTAM) (2010) has indicated that human exposure to Pb should be reduced and recommended to enhance the EFSA food category database. Overall, the present data may not only demonstrate a reduction of Pb concentrations in the organs of horses when compared to data 25 years ago, but may also expand the data pool on Pb concentrations in equine by-products.

The hepatic Cr concentrations did not differ when compared to the amounts in the renal cortex and medulla. Foals up to 1 year showed higher (137 (116–427) μg/kg) hepatic Cr concentrations than horses between 9–13 years (67.1 (22.0-118) μg/kg), and lower Cr concentrations were detected in the renal medulla of female horses when compared to male horses. The Sb concentrations in the liver of the horses were higher than in the renal cortex and medulla, while no gender- or age-dependencies were detected for the storage. In general, comparable Sb concentrations were measured in the liver and kidneys of humans (Sumino et al. 1975). Since little is known about the storage of Cr and Sb in the organism, further studies are needed to evaluate the metabolism of these elements and to explain the age- and gender-related differences for the storage of Cr in horses. However, the present data add valuable information on the Cr concentrations in organs of horses, which can be used for animal, but also for human nutrition. In particular, the EFSA Panel on Contaminants in the Food Chain (CONTAM) (2014) recently recommended to generate data on the Cr concentrations in food, why the present results may be relevant not only for diagnostics, but also for risk assessment.

## Conclusion

When compared to other animals, the measured amounts of Sr, Ba, Cd, Cu, Zn, Mn, Cr, Sb, Se and Pb in the livers and kidneys of horses often differed, possibly because of varying living or feeding conditions. The present data are of practical relevance, since horses could partially be considered as an indicator for the environmental pollution, and the equine liver and kidneys are edible animal by-products in the food chain. Interestingly, the measured concentrations of Sr, Ba, Cd, Cu, Zn, Mn, Cr, Sb, Se and Pb showed only small variations depending on the age or sex of the animals, which is in contrast to observations in other species. Future studies should evaluate the metabolism of the elements in the equine organism to explain the detected species-specific differences.

## Electronic supplementary material

Additional file 1: Table S1: Sex, age, breed and pathomorphological findings of the horses included in this study. (DOC 44 KB)
