# Supplementary material for: Concentrations of strontium, barium, cadmium, copper, zinc, manganese, chromium, antimony, selenium and lead in the equine liver and kidneys
Source: Springerplus. 2014 Jul 8;3:343. doi: 10.1186/2193-1801-3-343 (PMC4108716; doi:10.1186/2193-1801-3-343)
Supplement: Supplementary file 1 — Additional file 1: Table S1: Sex, age, breed and pathomorphological findings of the horses included in this study. (DOC 44 KB) [file 40064_2014_1053_MOESM1_ESM.doc]

**Additional file 1: Table S1:** Sex, age, breed and pathomorphological findings of the horses included in this study.

| **No.** | **Sex** | **Age** | **Breed** | **Pathomorphological findings** |
| --- | --- | --- | --- | --- |
| 1 | ♀ | 11 years | Tinker | intestinal infection with tapeworms (Anoplocephala perfoliata) |
| 2 | ♀ | 28 years | Warmblood | chronic intestinal nephritis and fibrosis; multifocal renal cysts |
| 3 | ♀ | 8 years | Warmblood | kidney fibrosis |
| 4 | ♀ | 16 years | Arabian | melanoma in the liver with multiple metastases; chronic intestinal nephritis |
| 5 | ♀ | 11 years | Thoroughbred | adrenocortical carcinoma |
| 6 | ♀ | 8 years | Warmblood | medullar calcification of the kidneys; chronic lymphocytic encephalitis |
| 7 | ♂ | 4 years | Warmblood | multifocal axonal degeneration and demyelination in the spinal cord region C6/7 |
| 8 | ♂ | 5 months | Thoroughbred | chronic enteritis |
| 9 | ♂ | 13 years | draft horse crossbreed | chromoproteinemic nephrosis |
| 10 | ♀ | 9 years | Warmblood | endocarditis |
| 11 | ♀ | 18 years | Pony | not specified (slaughtering) |
| 12 | ♀ | 20 years | Pony | not specified (slaughtering) |
| 13 | ♀ | 8 years | Haflinger crossbreed | not specified (slaughtering) |
| 14 | ♀ | 26 years | Warmblood | not specified (slaughtering) |
| 15 | ♀ | 16 years | Haflinger Arabian crossbreed | not specified (slaughtering) |
| 16 | ♂ | 2 years | Warmblood | not specified (slaughtering) |
| 17 | ♂ | 1 year | Warmblood | not specified (slaughtering) |
| 18 | ♂ | 13 years | Warmblood | not specified (slaughtering) |
| 19 | ♂ | 10 months | Shetland pony | not specified (slaughtering) |
| 20 | ♂ | 11 months | Shetland pony | not specified (slaughtering) |
| 21 | ♀ | 12 years | Pony | not specified (slaughtering) |
